# Using clinical prediction models to personalise lifestyle interventions for cardiovascular disease prevention: A systematic literature review

**DOI:** 10.1016/j.pmedr.2021.101672

**Published:** 2021-12-16

**Authors:** Anke Bruninx, Bart Scheenstra, Andre Dekker, Jos Maessen, Arnoud van 't Hof, Bas Kietselaer, Iñigo Bermejo

**Affiliations:** a Department of Radiation Oncology (MAASTRO), GROW School for Oncology and Developmental Biology, Maastricht University Medical Centre+, Maastricht, the Netherlands; b Department of Cardiothoracic Surgery, Maastricht University Medical Centre+, Maastricht, the Netherlands; c Cardiovascular Research Institute Maastricht (CARIM), Maastricht, the Netherlands; d Department of Cardiology, Maastricht University Medical Centre+, Maastricht, the Netherlands; e Department of Cardiology, Zuyderland Medical Centre, Heerlen, the Netherlands

**Keywords:** Cardiovascular diseases/prevention and control, Behavioral medicine, Decision support techniques, Models, cardiovascular, Patient-specific modeling

## Abstract

This study aimed to systematically review the use of clinical prediction models (CPMs) in personalised lifestyle interventions for the prevention of cardiovascular disease. We searched PubMed and PsycInfo for articles describing relevant studies published up to August 1, 2021. These were supplemented with items retrieved via screening references of citations and cited by references. In total, 32 studies were included. Nineteen different CPMs were used to guide the intervention. Most frequently, a version of the Framingham risk score was used. The CPM was used to inform the intensity of the intervention in five studies (16 %), and the intervention’s type in 31 studies (97 %). The CPM was supplemented with relative risk estimates for additional risk factors in three studies (9 %), and relative risk estimates for intervention effects in four (13 %). In addition to the estimated risk, the personalisation was determined using criteria based on univariable risk factors in 18 studies (56 %), a lifestyle score in three (9 %), and a physical examination index in one (3 %). We noted insufficient detail in reporting regarding the CPM’s use in 20 studies (63 %). In 15 studies (47 %), the primary outcome was a CPM estimate. A statistically significant effect favouring the intervention to the comparator arm was reported in four out of eight analyses (50 %), and a statistically significant improvement compared to baseline in five out of seven analyses (71 %). Due to the design of the included studies, the effect of the use of CPMs is still unclear. Therefore, we see a need for future research.

## Highlights

• The use of clinical prediction models (CPMs) to personalise interventions varies.
• We reviewed 32 CPM-guided lifestyle interventions to prevent cardiovascular disease.
• CPMs were mainly used to determine the intervention’s type, rarely its intensity.
• CPMs were supplemented with univariable risk factors, relative risk estimates, etc.
• Often reporting lacked detail and designs were unfit to assess the CPM’s impact.

## 1 Introduction

Cardiovascular disease (CVD) was the cause of an estimated 17.80 million deaths worldwide in 2017, according to the global burden of disease report published in 2018 (Roth et al., 2018). In comparison, the total number of deaths due to neoplasms (mainly cancer) was estimated at 9.56 million. Moreover, two CVDs, coronary artery disease and stroke, ranked respectively first and third in terms of estimated years of life lost (Roth et al., 2018).

An association between lifestyle, i.e. behavioural patterns, and CVD has been reported in several previous studies. For example, not smoking, increased fruit and vegetable intake, and increased leisure-time physical activity have been linked to less risk in meta-analyses (Aune et al., 2018, He et al., 2007, Sattelmair et al., 2011). Moreover, adhering to a healthy lifestyle, i.e. combining different health behaviours, has been reported to be associated with an estimated CVD risk reduction of 66 % (95 % CI: 59 – 72 %) via meta-analysis (Barbaresko et al., 2018).

Clinical prediction models (CPMs) estimate a diagnostic or prognostic clinical outcome based on relevant variables, such as patient characteristics, disease indicators, and treatment domains (Steyerberg, 2019). Due to the joint effects of separate risk factors, estimation of CVD risk using CPMs is deemed preferable to univariable approaches (Alderman, 1993, Jackson et al., 2005).

Well-validated CPMs have been endorsed by clinical guidelines. Examples are the systematic coronary risk evaluation (SCORE) estimator (Conroy et al., 2003, Piepoli et al., 2016, Systematic Coronary Risk Evaluation 2 Working Group and European Society of Cardiology Cardiovascular Risk Collaboration, 2021, Visseren et al., 2021) in Europe, the Framingham risk score (FRS) pooled cohort equations (PCE) (Arnett et al., 2019, Goff et al., 2014) in the United States of America, the QRISK estimator (Hippisley-Cox et al., 2008, Hippisley-Cox et al., 2017, National Institute for Health and Care Excellence, 2014) in the United Kingdom, and PREDICT in New Zealand (New Zealand Ministry of Health, 2018, Pylypchuk et al., 2018).

In addition to risk assessment and communication of this risk to the patient, these CPMs may be used to suggest an action to reduce CVD risk. After all, knowing the risk estimate does not change it, changing behaviours or medications do. Contrary to a one size fits all approach, the intervention may differ based on the CPM’s estimate, i.e. CPMs may be used to personalise the intervention.

A CPM may inform the intervention in a quantitative manner. People with a higher estimated risk may receive more intensive lifestyle support. For example, higher risk estimates may be linked to higher follow-up frequencies.

Additionally, one can imagine a CPM informs the intervention in a qualitative manner. For example, the CPM’s estimate for a particular participant may be adjusted to reflect hypothetical intervention effects. Those with the largest expected risk reductions may be given priority, if the patient and guiding physician are in accordance.

In this review, we focus on the use of CPMs to personalise lifestyle interventions for CVD prevention by suggesting an action. We aim to systematically review the literature to provide an overview of studies with such interventions, and analyse how CPMs are currently being used.

## 2 Methods

### 2.1 Study protocol registration

The study protocol was not registered.

### 2.2 Search strategy

We conducted a number of scoping searches for publications up to August 1, 2021 matching our inclusion criteria in the PubMed database and in the American Psychological Association PsycInfo database. We constructed search strings using various terms to capture three main concepts: CVD, a lifestyle-oriented prevention strategy, and prediction modelling. Table A1 in appendix A presents the search strings that led to at least one retained publication after screening based on title and abstract, which was conducted by a single reviewer (AB). The database searches were supplemented with items retrieved via non-systematic screening of references and cited by references. One reviewer (AB) did an initial screening based on full-text. If a publication was considered suitable to be included or when there were doubts whether to include or exclude a publication, a minimum of two reviewers read independently the full-text (AB, BS, IB). A final decision was made based on agreement between at least two reviewers. References of publications excluded based on full-text are provided in appendix B.

### 2.3 Eligibility criteria

The intervention described in the study had to focus on lifestyle change as a means for CVD prevention, and comprise a risk estimate of a CPM used as part of the intervention. Studies that used a risk estimate of a CPM exclusively as an eligibility criterion or to communicate risk were excluded. Studies that targeted specific patient populations other than people with CVD, such as people with diabetes, were excluded as well. All studies selected had to be described in a full-text publication in English. Finally, if multiple publications were derived from one unique intervention, we included only the main publication.

### 2.4 Data extraction and synthesis

Data extraction and synthesis was performed jointly by two reviewers (AB, IB).

The study name, citation, and region were extracted, and the study design was noted.

The following sample characteristics were extracted: the total number of participants at baseline, the type of population targeted, what age criterion for eligibility was applied, whether an inclusion criterion based on CVD risk was applied, and whether an exclusion criterion based on a history of CVD was applied. We summarised the lifestyle intervention described in the study, as well as the comparator, if the study was a randomised controlled trial (RCT). We classified the lifestyle intervention domains as related to smoking, physical activity, nutrition, alcohol intake, psychological well-being, medication adherence, or other. Moreover, we noted whether medications were treatment options, including those via referral.

In addition, we extracted what CPM was used in the intervention, noted which data elements, if any, were used to supplement the CPM estimate, and classified whether the suggested action based on the CPM was to determine the lifestyle intervention’s intensity and / or its type. The lifestyle intervention’s intensity refers to quantitative differences, for example, a higher follow-up frequency for those at higher risk. The lifestyle intervention’s type refers to qualitative suggestions, such as indicating which lifestyle domain is associated with most gains in terms of risk reduction. Note that we are considering suggestions based on the CPM. These are not necessarily carried forward in the actual treatment plan, for example, if smoking cessation would be preferable according the CPM, but the participant does not wish to quit smoking. Furthermore, we indicated whether reporting lacked detail concerning the description of the CPM and its use. Arguments are provided in appendix C.

Lastly, we extracted the length of follow-up, classified the primary outcome(s) as related to mortality, morbidity, CPM estimate, biomarkers (blood pressure, lipids, other), anthropometrics, lifestyle (smoking, physical activity, nutrition, other), medications, or other, and noted the results.

Frequencies and proportions were tabulated when appropriate to provide an overview.

### 2.5 Reporting

Reporting followed guidance provided by the preferred reporting items for systematic reviews and meta-analyses (PRISMA) statement (Page et al., 2021). The completed checklist for abstracts and main text are provided in appendix D.

## 3 Results

### 3.1 Search results

**Fig. 1:**
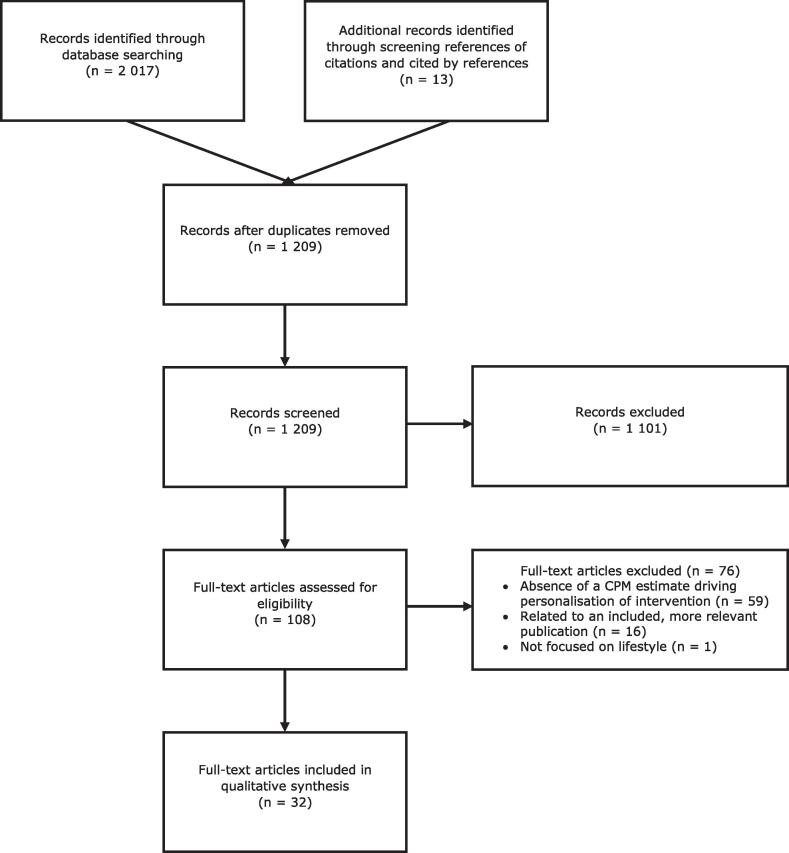
PRISMA flow-diagram.

Fig. 1 presents an overview of the selection process, based on the preferred reporting items for systematic reviews and meta-analyses (PRISMA) flow-diagram template (Liberati et al., 2009). There were 2 017 items identified via database searches, and 13 by screening references of citations and cited by references. In total, there were 1 209 unique items screened. There were 1 101 items excluded based on title and abstract, and 108 were selected for a more in-depth assessment. Based on full-text, we excluded an additional 76 items: 59 due to the absence of a CPM estimate linked to a suggested action in the intervention, 16 due to their relation with an included, more relevant publication, and one which was not focused on lifestyle (references are provided in appendix B). This final selection led to a total of 32 included publications.

### 3.2 Data extraction and synthesis

Table 1 provides the following information per study: study name (if any), citation, region, study design, participants, a description of the intervention and comparator (if the study design was a RCT), a description of aspects concerning the CPM used in the intervention, and a summary regarding the primary outcome(s). Publications are listed chronologically, ranging from 1994 to 2020. Seventeen (53 %) studies were located in Europe, ten (31 %) in North America, three (9 %) in Oceania, and two (6 %) in Asia. Regarding the study design, 20 out of 32 studies (63 %) were RCTs and 12 (38 %) were single-arm studies.

**Table 1:** Synthesis: study name (if any), citation, region, study design, participants, intervention and comparator (if RCT), CPM, supplement, and use of CPM, primary outcome(s).

	**Study name (if any)**	**Citation**	**Region**	**Study design**	**Participants**	**Intervention and comparator (if RCT)**	**CPM, supplement, and use of CPM**	**Primary outcome(s)**
**1.**	British FHS	(Wood et al., 1994)	UK	RCT	▪ n = 12 472 ▪ Primary care ▪ Males 40 – 59 years and their spouses	▪ Risk assessment and communication, lifestyle counselling concerning smoking, physical activity, nutrition, and / or alcohol intake, diary, brochures, frequency of follow-up according to degree of risk, referrals, medications possible via referral ▪ TAU, same practice and matched practice	▪ British FHS risk score (Thompson et al., 1996) ▪ Supplemented with univariable risk factors ▪ Suggested action: determine intervention intensity	▪ Length of follow-up: 1 year ▪ CPM estimate, biomarkers (blood pressure, lipids, other: glucose), anthropometrics, lifestyle (smoking) ▪ All outcomes, except glucose and lipids in female group, statistically significant differences in favour of the intervention; concerning glucose and lipids in female group, no statistically significant differences
**2.**	–	(Ketola et al., 2001)	FI	RCT	▪ n = 150 ▪ Primary care ▪ 18 – 65 years ▪ Inclusion based on CVD risk	▪ Personal health plan according to risk, lifestyle changes concerning physical activity, and / or nutrition, medications, two scheduled follow-up moments between baseline and end-point with doctor and nurse ▪ TAU, brochure	▪ North Karelia risk score (modified) (Suomen Sydäntautiliitto et al., 1986) ▪ Suggested action: determine intervention type ▪ Insufficient detail in reporting	▪ Length of follow-up: 2 years ▪ CPM estimate ▪ No statistically significant difference
**3.**	–	(Edelman et al., 2006)	USA	RCT	▪ n = 154 ▪ Primary care ▪ ≥ 45 years ▪ Inclusion based on CVD risk ▪ Exclusion based on CVD	▪ Risk assessment and communication, personal health plan: education, individual and group counselling concerning smoking, physical activity, nutrition, psychological well-being, and / or other (e.g., communication skills) ▪ TAU, risk assessment and communication	▪ Know your number (Hu and Root, 2005) ▪ Suggested action: determine intervention type ▪ Insufficient detail in reporting	▪ Length of follow-up: 10 months ▪ CPM estimate ▪ Statistically significant steeper improvement in favour of the intervention
**4.**	Hartslag Limburg	(Harting et al., 2006)	NL	RCT	▪ n = 1 300 ▪ Primary care ▪ Inclusion based on CVD risk	▪ Optimisation of medical treatment and health counselling concerning smoking, physical activity, nutrition, and / or medication adherence, referrals ▪ TAU	▪ Coronary risk charts (Wood et al., 1998): FRS Anderson (Anderson et al., 1991) ▪ Supplemented with univariable risk factors ▪ Suggested action: determine intervention type ▪ Insufficient detail in reporting	▪ Length of follow-up: 1.5 years ▪ Not specified
**5.**	Simon Fraser heart health report card system	(Wister et al., 2007)	CA	RCT	▪ n = 611 ▪ General population ▪ 45 – 64 years ▪ Inclusion based on CVD risk	▪ Report card sent to participant and participant’s GP, telehealth counselling concerning smoking, physical activity, nutrition, and / or psychological well-being, educational materials ▪ TAU	▪ FRS Wilson (Wilson et al., 1998) ▪ Supplemented with univariable risk factors ▪ Suggested action: determine intervention type ▪ Insufficient detail in reporting	▪ Length of follow-up: 1 year ▪ CPM estimate ▪ Statistically significant difference in favour of the intervention in the primary prevention group, not in the secondary prevention group
**6.**	ARRIBA-Herz	(Krones et al., 2008)	DE	RCT	▪ n = 1 132 ▪ Primary care	▪ Decision-aid, personal health plan concerning smoking, physical activity, nutrition, and / or medications, training sessions for physicians ▪ TAU, unrelated training sessions for physicians	▪ ARRIBA-Herz (Krones et al., 2008): FRS ATP III (National Cholesterol Education Program Expert Panel, 2002) ▪ Supplemented with relative risk estimates concerning additional risk factors and intervention effects ▪ Suggested action: determine intervention type ▪ Insufficient detail in reporting	▪ Length of follow-up: 6 months ▪ CPM estimate, other (patient activation, decisional regret) ▪ No statistically significant difference CPM estimates, statistically significant improved patient activation (patient’s participation / satisfaction) and less decisional regret in favour of the intervention
**7.**	Inter99 study	(Jørgensen et al., 2014)	DK	RCT	▪ n = 59 616 ▪ General population ▪ Near age 30, 35, 40, 45, 50, 55, 60 years ▪ Inclusion based on CVD risk	▪ High intensity condition: risk assessment and communication, individual counselling concerning smoking, physical activity, nutrition, and / or alcohol intake, brochures, group-based counselling for high risk participants concerning smoking, physical activity, and / or nutrition; low intensity condition: risk assessment and communication, individual counselling concerning smoking, physical activity, nutrition, and / or alcohol intake, brochures, referrals, medications possible via referral (Jørgensen et al., 2003) ▪ TAU	▪ Copenhagen risk score (Thomsen et al., 2001) ▪ Supplemented with univariable risk factors ▪ Suggested action: determine intervention type	▪ Length of follow-up: 10 years ▪ Mortality / morbidity (composite fatal / non-fatal IHD) ▪ No statistically significant differences comparing intervention and comparator condition
**8.**	Healthy hearts	(Richardson et al., 2008)	UK	Single-arm study	▪ n = 596 ▪ Primary care ▪ 45 – 64 years ▪ Exclusion based on CVD	▪ Risk assessment and communication, advice, personal risk mitigation via referrals concerning primary care, smoking, physical activity, and / or nutrition, medications possible via referral	▪ FRS Anderson (Anderson et al., 1991) ▪ Supplemented with univariable risk factors ▪ Suggested action: determine intervention type ▪ Insufficient detail in reporting	▪ Length of follow-up: 1 year ▪ CPM estimate ▪ Statistically significant improvement from baseline
**9.**	REACH OUT	(Benner et al., 2008)	EU	RCT	▪ n = 1 103 ▪ Primary care ▪ 45 – 64 years ▪ Inclusion based on CVD risk ▪ Exclusion based on CVD	▪ Risk assessment and communication, personal health plan concerning smoking, physical activity, nutrition, and / or medication adherence, medications, three follow-ups by phone, training for physicians (Benner et al., 2007) ▪ TAU	▪ FRS ATP III (National Cholesterol Education Program Expert Panel, 2002) ▪ Supplemented with univariable risk factors ▪ Suggested action: determine intervention type	▪ Length of follow-up: 6 months ▪ CPM estimate ▪ Statistically significant difference in favour of the intervention
**10.**	IMPALA	(Koelewijn-van Loon et al., 2009)	NL	RCT	▪ n = 615 ▪ Primary care ▪ Smoking and age ≥ 50 years for males, smoking and age ≥ 55 years for females (non-smokers at high risk without any age restriction were included as well) ▪ Inclusion based on CVD risk ▪ Exclusion based on CVD	▪ Risk assessment and communication, decision-aid, adapted motivational interviewing by nurses concerning smoking, physical activity, nutrition, alcohol intake, and / or medication adherence, medications, training for nurses, referrals (Koelewijn-van Loon et al., 2008) ▪ TAU, training for nurses (less extensive)	▪ SCORE (Conroy et al., 2003) / UKPDS (Stevens et al., 2001) ▪ Suggested action: determine intervention type ▪ Insufficient detail in reporting	▪ Length of follow-up: 1 year ▪ Not specified
**11.**	COHRT	(Nolan et al., 2011)	CA	RCT	▪ n = 680 ▪ General population ▪ 35 – 74 years ▪ Inclusion based on CVD risk	▪ Risk assessment and communication to participant and participant’s GP, advice concerning smoking, physical activity, and / or nutrition, brochures, referrals to community programs ▪ Same as above plus one hour telehealth counselling concerning smoking, physical activity, and / or nutrition for 6 weeks	▪ FRS Wilson (Wilson et al., 1998) ▪ Suggested action: determine intervention type ▪ Insufficient detail in reporting	▪ Length of follow-up: 6 months ▪ Lifestyle (physical activity, nutrition) ▪ Statistically significant differences in favour of the intervention concerning physical activity and nutrition
**12.**	HAPPY / MyCLIC: NL	(Hofstra et al., 2011, Yousuf et al., 2019)	NL	Single-arm study	▪ n = 595 ▪ General population	▪ Risk assessment and communication, lifestyle counselling via e-mail concerning smoking, physical activity, and nutrition, referrals, medications possible via referral	▪ PROCAM (Assmann et al., 2002) ▪ Supplemented with lifestyle score ▪ Suggested action: determine intervention type ▪ Insufficient detail in reporting	▪ Length of follow-up: 3 months ▪ Not specified
**13.**	Heart to heart	(Sheridan et al., 2011)	USA	RCT	▪ n = 160 ▪ 40 – 79 years ▪ Internal medicine practice patients ▪ Inclusion based on CVD risk ▪ Exclusion based on CVD	▪ Risk assessment and communication, decision-aid including selecting smoking cessation and / or medications to mitigate risk, three e-mails to enhance adherence, education session for physicians ▪ TAU	▪ FRS Wilson (Wilson et al., 1998) ▪ Supplemented with relative risk estimates concerning intervention effects ▪ Suggested action: determine intervention type	▪ Length of follow-up: 3 months ▪ Not specified
**14.**	Fremantle primary prevention study	(Brett et al., 2012)	AU	RCT	▪ n = 1 200 ▪ Primary care ▪ 40 – 80 years ▪ Exclusion based on CVD	▪ Risk assessment and communication, five GP visits, including at baseline and at end of study, counselling concerning smoking, physical activity, and / or nutrition, medications, referrals ▪ TAU, GP visit at baseline and at end of study	▪ New Zealand cardiovascular risk calculator (New Zealand Guidelines Group, 2003): FRS Anderson (modified) (Anderson et al., 1991) ▪ Supplemented with univariable risk factors ▪ Suggested action: determine intervention type ▪ Insufficient detail in reporting	▪ Length of follow-up: 1 year ▪ CPM estimate ▪ No statistically significant difference
**15.**	PreCardio	(Claes et al., 2013)	BE	RCT	▪ n = 314 ▪ Self-employed lawyers ▪ 25 – 75 years	▪ Medical programme: risk assessment and communication concerning smoking, physical activity, nutrition, and / or medications, referrals ▪ Medical and lifestyle programme: same as above plus individual coaching concerning smoking, physical activity, and / or nutrition, website tailored to the participant’s profile	▪ SCORE (Conroy et al., 2003) ▪ Supplemented with univariable risk factors ▪ Suggested action: determine intervention intensity, determine intervention type	▪ Length of follow-up: 3 years ▪ Biomarkers (blood pressure, lipids), anthropometrics ▪ No statistically significant differences
**16.**	ANCHOR	(Cox et al., 2013)	CA	Single-arm study	▪ n = 1 509 ▪ Primary care ▪ ≥ 30 years ▪ Inclusion based on CVD risk (analysis) ▪ Exclusion based on CVD (analysis)	▪ Risk assessment and communication, lifestyle counselling concerning smoking, physical activity, nutrition, psychological well-being, medication adherence, and / or other (focus on acquiring behavioural change skills), training for counsellors, referrals, medications possible via referral (Cox et al., 2011)	▪ FRS ATP III (National Cholesterol Education Program Expert Panel, 2002) ▪ Suggested action: determine intervention intensity, determine intervention type ▪ Supplemented with univariable risk factors	▪ Length of follow-up: 1 year ▪ CPM estimate ▪ Statistically significant improvement from baseline
**17.**	Heart to health	(Keyserling et al., 2014)	USA	RCT	▪ n = 385 ▪ Primary care ▪ 35 – 79 years ▪ Inclusion based on CVD risk ▪ Exclusion based on CVD	▪ Decision-aid, selecting treatment options guided by risk estimates concerning smoking, physical activity, nutrition, and / or medications, medication adherence, seven counselling sessions via web, referrals ▪ Same as above but seven counselling sessions by health counsellor instead of via web	▪ FRS Anderson (Anderson et al., 1991) ▪ Supplemented with relative risk estimates concerning intervention effects ▪ Suggested action: determine intervention type	▪ Length of follow-up: 1 year ▪ CPM estimate ▪ Specification of within condition analyses at 4 months as primary outcomes, statistically significant improvement from baseline in both conditions
**18.**	My family medical history and me (pilot)	(Imes et al., 2015)	USA	Single arm study (pilot)	▪ n = 15 ▪ University students ▪ 18 – 25 years ▪ Inclusion based on CVD risk ▪ Exclusion based on CVD	▪ Risk assessment and communication focused on family history of CVD, lifestyle advice concerning smoking, physical activity, and nutrition, brochures	▪ FRS ATP III (National Cholesterol Education Program Expert Panel, 2002) ▪ Supplemented with univariable risk factors ▪ Suggested action: determine intervention type	▪ Length of follow-up: 2 weeks ▪ Not specified
**19.**	Protecting healthy hearts program / GARDIAN	(Carrington and Stewart, 2015)	AU	Single-arm study	▪ n = 530 ▪ Population living remotely from health services ▪ ≥ 18 years	▪ Risk assessment and communication, depending on risk category one follow-up moment by phone and one or two follow-up moments in person, personal health plan concerning smoking, physical activity, nutrition, alcohol intake, psychological well-being, and / or medication adherence, brochures, referrals, medications possible via referral, training for diabetes educators / nurses (Carrington et al., 2012)	▪ Absolute CVD risk score (Carrington et al., 2012): FRS Anderson (Anderson et al., 1991) ▪ Supplemented with univariable risk factors ▪ Suggested action: determine intervention intensity, determine intervention type	▪ Length of follow-up: 6 months ▪ Not specified
**20.**	–	(Abbas et al., 2015)	UK	Single-arm study	▪ n = 635 ▪ Local governmental employees in deprived areas ▪ ≥ 40 years ▪ Exclusion based on CVD	▪ Risk assessment and communication, possible referrals concerning smoking, physical activity, nutrition, alcohol intake, psychological well-being, and / or medications	▪ National health services health check’s cardiovascular risk assessor (National Health Services Health Check Programme, 2009): QRISK2 (Hippisley-Cox et al., 2008) / FRS Anderson (Anderson et al., 1991) ▪ Supplemented with univariable risk factors ▪ Suggested action: determine intervention type	▪ Length of follow-up: 9 months ▪ Not specified
**21.**	IEHPS (pilot)	(Liu et al., 2015)	CN	RCT (pilot)	▪ n = 589 ▪ Healthcare employees ▪ 45 – 75 years ▪ Exclusion based on CVD	▪ Risk assessment (including physical examination) and communication, counselling concerning smoking, physical activity, nutrition, alcohol intake, and / or psychological well-being, medications, educational handbook, follow-up phone calls and text messages ▪ TAU, physical examination	▪ CPM for ICVD (Wu et al., 2006) ▪ Supplemented with univariable risk factors, physical examination index ▪ Suggested action: determine intervention intensity, determine intervention type ▪ Insufficient detail in reporting	▪ Length of follow-up: 1 year ▪ CPM estimate ▪ Statistically significant difference between intervention and comparator in favour of intervention
**22.**	INTEGRATE (pilot)	(van den Brekel-Dijkstra et al., 2016)	NL	Single-arm study (pilot)	▪ n = 230 ▪ Primary care ▪ 45 – 70 years ▪ Exclusion based on CVD	▪ Stepped risk assessment, risk communication, decision-aid, lifestyle advice via online tool concerning smoking, physical activity, nutrition, alcohol intake, and / or psychological well-being, medications, referrals	▪ SCORE (modified) (Van der Kalken and Kraaijenhagen, 2007) ▪ Supplemented with univariable risk factors ▪ Suggested action: determine intervention type ▪ Insufficient detail in reporting	▪ Length of follow-up: 6 months ▪ Not specified
**23.**	–	(Siren et al., 2016)	FI	Single-arm study	▪ n = 185 ▪ Males aged 40 years living in Helsinki ▪ Inclusion based on CVD risk (analysis)	▪ Risk assessment and communication, lifestyle counselling, brochures concerning smoking, physical activity, and nutrition, referrals, medication possible via referral	▪ North Karelia risk score (modified) (Suomen Sydäntautiliitto et al., 1986) ▪ Supplemented with univariable risk factors ▪ Suggested action: determine intervention type ▪ Insufficient detail in reporting	▪ Length of follow-up: 5 years ▪ CPM estimate, lifestyle (smoking, physical activity, nutrition) (stratification by follow-up care: none, primary care, occupational health care) ▪ Statistically significant improvements concerning one of two CPM estimates (non-significant: p = 0.060) (some statistically significant differences between follow-up care groups), statistically significant improvements concerning smoking, physical activity, and nutrition (no statistically significant differences between follow-up care groups)
**24.**	CHARLAR	(Krantz et al., 2017)	USA	Single-arm study	▪ n = 1 099 ▪ Latin-Americans ▪ ≥ 45 years	▪ Risk assessment and communication, 3 months prevention program concerning smoking, physical activity, nutrition, and other (e.g., goal setting), education, referrals, medications possible via referral	▪ FRS PCE (Goff et al., 2014) ▪ Supplemented with univariable risk factors ▪ Suggested action: determine intervention type ▪ Insufficient detail in reporting	▪ Length of follow-up: 3 months ▪ CPM estimate ▪ Statistically significant improvement from baseline
**25.**	DECADE	(Tinsel et al., 2018)	DE	RCT (pilot)	▪ n = 87 ▪ Primary care ▪ Inclusion based on CVD risk	▪ Decision-aid, personal health plan concerning smoking, physical activity, and / or nutrition, medications, four follow-up sessions, brochures ▪ Same as above minus brochures	▪ ARRIBA-Herz (Krones et al., 2008): FRS ATP III (National Cholesterol Education Program Expert Panel, 2002) ▪ Supplemented with relative risk estimates concerning risk factors and intervention effects ▪ Suggested action: determine intervention type ▪ Insufficient detail in reporting	▪ Length of follow-up: 4 months ▪ Other (patient activation) ▪ Statistically significant improvement in favour of the intervention
**26.**	ACTIVATE	(Oddone et al., 2018)	USA	RCT	▪ n = 417 ▪ USA veterans ▪ Inclusion based on CVD risk	▪ Online risk assessment and communication, two phone calls by health coach, referrals concerning smoking, physical activity, and / or nutrition ▪ Online risk assessment and communication	▪ MyHealtheVet’s health age (Martin and Sartori, 2014) ▪ Suggested action: determine intervention type ▪ Insufficient detail in reporting	▪ Length of follow-up: 6 months ▪ Other (patient activation) ▪ Statistically significant improvement in patient activation (enrolment in prevention program) in favour of the intervention
**27.**	HAPPY / MyCLIC: AZM	(Yousuf et al., 2019)	NL	Single-arm study	▪ n = 1 062 ▪ Healthcare employees ▪ Exclusion based on CVD	▪ Risk assessment and communication, lifestyle counselling concerning smoking, physical activity, nutrition, alcohol intake, psychological well-being, and / or other (e.g., sleep) via online tool and e-mail	▪ PROCAM (Assmann et al., 2002) ▪ Supplemented with a lifestyle score ▪ Suggested action: determine intervention type ▪ Insufficient detail in reporting	▪ Length of follow-up: 1 year ▪ Not specified
**28.**	HAPPY / MyCLIC: London	(Khanji et al., 2019, Yousuf et al., 2019)	UK	RCT	▪ n = 402 ▪ Primary care ▪ 40 – 74 years ▪ Inclusion based on CVD risk ▪ Exclusion based on CVD	▪ Risk / lifestyle assessment and communication, counselling by physician, lifestyle counselling concerning smoking, physical activity, nutrition, alcohol intake, psychological well-being, and / or other (e.g., sleep) via online tool, e-mail reminders ▪ TAU, counselling by physician	▪ QRISK2 (Hippisley-Cox et al., 2008) ▪ Supplemented with a lifestyle score ▪ Suggested action: determine intervention type ▪ Insufficient detail in reporting	▪ Length of follow-up: 6 months ▪ Biomarkers (other: carotid-femoral pulse wave velocity) ▪ No statistically significant difference intervention and comparator
**29.**	–	(Kavita et al., 2020)*	IN	Single-arm study	▪ n = 402 ▪ Tertiary care ▪ ≥ 40 years ▪ Inclusion based on CVD risk ▪ Exclusion based on CVD	▪ Risk assessment and communication, education and counselling concerning smoking, physical activity, nutrition, alcohol intake, and / or medication adherence by trained nurses, three follow-up phone calls (Kavita et al., 2018)	▪ WHO / ISH (World Health Organization, 2007) ▪ Supplemented with univariable risk factors ▪ Suggested action: determine intervention type ▪ Insufficient detail in reporting	▪ Length of follow-up: 1 year ▪ CPM estimate ▪ Statistically significant improvements from baseline
**30.**	INTEGRATE	(Stol et al., 2020)	NL	RCT	▪ n = 1 934 ▪ Primary care ▪ 45 – 70 years ▪ Exclusion based on CVD	▪ Stepped risk assessment, risk communication, lifestyle advice online or treatment by GP based on risk including addressing smoking, physical activity, and / or nutrition, medications, referrals (Badenbroek et al., 2014, Badenbroek et al., 2016) ▪ TAU	▪ CPM for CMD (Alssema et al., 2012) / SCORE (Conroy et al., 2003) ▪ Supplemented with univariable risk factors ▪ Suggested action: determine intervention type	▪ Length of follow-up: 1 year ▪ CPM estimate, biomarkers (blood pressure, lipids, other: glucose), anthropometrics, lifestyle (smoking), medications, other (CMD detection) ▪ Within intervention condition analyses: statistically significant improvement from baseline CPM estimate, only after adjustment for aging, statistically significant improvements concerning blood pressure and lipids, no statistically significant improvement concerning glucose, mixed results concerning anthropometrics (statistically significant improvement concerning waist circumference, not BMI), no statistically significant reduction in percentage of smokers from baseline; between conditions analyses: statistically significant increased detection CMD and more prescriptions of CMD medications in intervention condition
**31.**	CONNECT	(Redfern et al., 2020)	AU	RCT	▪ n = 934 ▪ Primary care ▪ ≥ 18 years ▪ Inclusion based on CVD risk	▪ Access to application with electronic health record connection displaying current diagnoses and medications, educational materials, risk calculator, lifestyle change support concerning smoking, physical activity, nutrition, psychological well-being, and / or medication adherence, medications possible via encouragement discussion with GP, social media, possibility to receive additional advice via e-mail and / or SMS, support service ▪ TAU	▪ FRS Anderson (Anderson et al., 1991) ▪ Suggested action: determine intervention type ▪ Insufficient detail in reporting	▪ Length of follow-up: 1 year ▪ Lifestyle (other: medication adherence) ▪ No statistically significant difference
**32.**	–	(Kwon et al., 2020)	USA	Single-arm study	▪ n = 38 ▪ Weight management clinic patients ▪ ≥ 18 years ▪ Inclusion based on CVD risk	▪ Access to application to track and improve nutrition	▪ Healthy heart score (Chiuve et al., 2014) ▪ Suggested action: determine intervention type	▪ Length of follow-up: 5 weeks ▪ Not specified

ACTIVATE = a coaching by telephone intervention for veterans and care team engagement; ANCHOR = a novel approach to cardiovascular health by optimizing risk management; ARRIBA-Herz = Aufgabe gemeinsam definieren, Risiko subjektiv, Risiko objektiv, Information über Präventionsmöglichkeiten, Bewertung der Präventionsmöglichkeiten und Absprache über weiteres Vorgehen – Herz (define task together, subjective risk, objective risk, information about prevention options and agreement on further action - heart); ATP = adult treatment panel; AU = Australia; AZM = Academisch Ziekenhuis Maastricht (Maastricht University Medical Centre +); BE = Belgium; BMI = body mass index; CA = Canada; CHARLAR = community heart health actions for Latinos at risk; CMD = cardiometabolic disease; CN = China; COHRT = community outreach heart health and risk reduction trial; CONNECT = consumer navigation of electronic cardiovascular tools; CPM = clinical prediction model; CVD = cardiovascular disease; DE = Deutschland (Germany); DECADE = decision-aid, action planning, and follow-up support for patients to reduce the 10-year risk of CVD; DK = Denmark; EU = European Union; FHS = family heart study; FI = Finland; FRS = Framingham risk score; GARDIAN = green, amber, red delineation of risk and need; GP = general practitioner; HAPPY = heart attack prevention program for you; ICVD = ischemic cardiovascular disease; IEHPS = individualised electronic healthcare prescription software; IHD = ischemic heart disease; IMPALA = improving patient adherence to lifestyle advice; IN = India; MyCLIC = my cardiac lifestyle intervention coach; NL = Netherlands; PCE = pooled cohort equations; PROCAM = prospective cardiovascular Münster study; RCT = randomised controlled trial; REACH OUT = risk evaluation and communication health outcomes and utilization trial; SCORE = systematic coronary risk evaluation; SMS = short message service; TAU = Treatment as usual; UK = United Kingdom; UKPDS = United Kingdom prospective diabetes study; USA = United States of America; WHO / ISH = World Health Organization / International Society of Hypertension.

* This publication described two studies, the second did not satisfy our eligibility criteria, and was consequently not included.

Table 2, Table 3, Table 4 summarise different aspects of the studies. Table 2 presents an overview of the participants targeted by the studies, intervention domains possible to select, and the use of decision-aids. The total number of participants of the reviewed studies ranged from 15 to 59 616. Most studies targeted primary care patients (50 %), the general population (13 %), or groups based on the participant’s occupation (13 %). In the majority of the studies (72 %), an eligibility criterion based on age (excluding specifying an age ≥ 18 years) was applied. Furthermore, in 59 % of the studies, potential participants were included if they had a high CVD risk. Additionally, in half of the studies (50 %), potential participants with known CVD were excluded. Most studies included multiple intervention domains, and smoking cessation, physical activity, and nutrition were included in more than 90 % of studies. About one-third of the interventions included alcohol intake, psychological well-being, and / or medication adherence. Less frequently, other domains, such as sleep or goal setting, were considered. In the majority of the interventions (72 %), medications were considered. Decision-aids were used in six interventions (19 %).

**Table 2:** Summary of study participants, intervention domains, and decision-aids.

			**n (%)**
**Participants**	**Target population**	**Primary care patients**	16 (50 %)
		**General population**	4 (13 %)
		**Based on occupation**	4 (13 %)
		**Other**	8 (25 %)
	**Eligibility criteria**	**Age**	23 (72 %)
		**Inclusion based on CVD risk**	19 (59 %)
		**Exclusion based on CVD**	16 (50 %)
**Intervention domains**	**Lifestyle**	**Smoking**	30 (94 %)
		**Physical activity**	30 (94 %)
		**Nutrition**	31 (97 %)
		**Alcohol intake**	10 (31 %)
		**Psychological well-being**	10 (31 %)
		**Medication adherence**	9 (28 %)
		**Other**	5 (16 %)
	**Medications**		23 (72 %)
**Decision-aids**			6 (19 %)
**Total number of studies**			32 (100 %)

CVD = cardiovascular disease.

**Table 3:** Breakdown of the CPMs used in each study, how they were supplemented and used, and whether reporting was lacking detail.

		**n (%)**
**Name**	**British FHS risk score** (Thompson et al., 1996)	1 (3 %)
	**Copenhagen risk score (**Thomsen et al., 2001**)**	1 (3%)
	**CPM for CMD (**Alssema et al., 2012**)**	1 (3 %)
	**CPM for ICVD (**Wu et al., 2006**)**	1 (3%)
	**FRS Anderson (**Anderson et al., 1991**)**	5 (16 %)
	**FRS Anderson (modified) (**New Zealand Guidelines Group, 2003**)**	1 (3 %)
	**FRS ATP III (**National Cholesterol Education Program Expert Panel, 2002**)**	5 (16 %)
	**FRS Wilson (**Wilson et al., 1998**)**	3 (9 %)
	**FRS PCE (**Goff et al., 2014**)**	1 (3 %)
	**Healthy heart score (**Chiuve et al., 2014**)**	1 (3 %)
	**Know your number (**Hu and Root, 2005**)**	1 (3 %)
	**MyHealtheVet’s health age (**Martin and Sartori, 2014**)**	1 (3 %)
	**North Karelia risk score (modified) (**Suomen Sydäntautiliitto et al., 1986**)**	2 (6 %)
	**SCORE (**Conroy et al., 2003**)**	4 (13 %)
	**SCORE (modified) (**Van der Kalken and Kraaijenhagen, 2007**)**	1 (3 %)
	**PROCAM (**Assmann et al., 2002**)**	2 (6 %)
	**QRISK2 (**Hippisley-Cox et al., 2008**)**	2 (6 %)
	**UKPDS (**Stevens et al., 2001**)**	1 (3 %)
	**WHO / ISH (**World Health Organization, 2007**)**	1 (3 %)
**Supplement**	**Univariable risk factors**	18 (56 %)
	**Relative risk estimates for additional risk factor (s)**	3 (9 %)
	**Relative risk estimates for intervention effect (s)**	4 (13 %)
	**Lifestyle score**	3 (9 %)
	**Physical examination index**	1 (3 %)
**Use**	**Determine intervention intensity**	5 (16 %)
	**Determine intervention type**	31 (97 %)
**Insufficient detail in reporting**		20 (63 %)
**Total number of studies**		32 (100 %)

ATP = adult treatment panel; CMD = cardiometabolic disease; CPM = clinical prediction model; FHS = family heart study; FRS = Framingham risk score; ICVD = ischemic cardiovascular disease; PCE = pooled cohort equations; PROCAM = prospective cardiovascular Münster study; SCORE = systematic coronary risk evaluation; UKPDS = United Kingdom prospective diabetes study; WHO / ISH = World Health Organization / International Society of Hypertension.

**Table 4:** Summary of reported statistically significant effects in favour of the intervention concerning the primary outcomes.

			**Intervention versus comparator**	**Comparison to baseline**
			**n (%)**	**n (%)**
**Mortality / morbidity**			0 / 1 (0 %)	–
**CPM estimate**			4 / 8 (50 %)	5 / 7 (71 %)
**Biomarkers**	**Blood pressure**		1 / 2 (50 %)	1 / 1 (100 %)
	**Lipids**		0 / 2 (0 %)	1 / 1 (100 %)
	**Other**	**Glucose**	0 / 1 (0 %)	0 / 1 (0 %)
		**Carotid-femur pulse wave velocity**	0 / 1 (0 %)	–
**Anthropometrics**			1 / 2 (50 %)	0 / 1 (0 %)
**Lifestyle**	**Smoking**		1 / 1 (100 %)	1 / 2 (50 %)
	**Physical activity**		1 / 1 (100 %)	1 / 1 (100 %)
	**Nutrition**		1 / 1 (100 %)	1 / 1 (100 %)
	**Other**	**Medication adherence**	0 / 1 (0 %)	–
		**Patient activation**	3 / 3 (100 %)	–
**Medications**			1 / 1 (100 %)	–
**Other**	**Detection CMD**		1 / 1 (100 %)	–
	**Decisional regret**		1 / 1 (100 %)	–

CMD = cardiometabolic disease; CPM = clinical prediction model.

Table 3 presents a breakdown of the CPMs used in the studies, as well as how they were used to personalise the interventions, and whether and how supplementary sources were used in the personalisation. Fifteen studies (47 %) reported using a version of the FRS (Anderson et al., 1991, Goff et al., 2014, National Cholesterol Education Program Expert Panel, 2002, New Zealand Guidelines Group, 2003, Wilson et al., 1998), while a version of the SCORE estimator (Conroy et al., 2003, Van der Kalken and Kraaijenhagen, 2007) was used in five (16 %). The North Karelia risk score (modified) (Suomen Sydäntautiliitto et al., 1986), prospective cardiovascular Münster study (PROCAM) estimator (Assmann et al., 2002), and QRISK2 (Hippisley-Cox et al., 2008) were each used twice (6 %). The other CPMs were used only in one study each (3 %). The CPM was most often, in 18 interventions (56 %), supplemented with univariable risk factors. For example, part of the intervention described by Abbas et al. (2015) consisted of a referral to a weight management specialist only if the participant had a CPM estimate ≥ 10 % as well as a BMI ≥ 28. Three studies (9%) reported applying relative risk estimates to the risk estimated by the CPM to account for additional risk factors not included in the CPM. For example, Cox et al. (2013) reported multiplying the CPM’s estimate by two if the participant had relatives with premature CVD. Four studies (13 %) reported using relative risk estimates from literature to calculate the expected reduction in risk. For example, in the decision-aid included as a supplementary material by Krones et al. (2008), a relative risk reduction of approximately 35 % for quitting smoking was reported. The lifestyle score was used in three related interventions (9 %), part of the heart attack prevention program for you (HAPPY) / my cardiac lifestyle intervention coach (MyCLIC). Reporting lacked detail to understand thoroughly how this lifestyle score was constructed and used. Lastly, the use of a physical examination index was reported once (3 %), by Liu et al. (2015). No definition was provided. Regarding the use of the CPM, we categorised five (16 %) interventions as using the CPM in a quantitative manner to determine the intervention’s intensity, and 31 (97 %) in a qualitative manner to determine the intervention’s type. Finally, we deemed reporting concerning the use of the CPM lacked detail in 20 studies (63 %) (argumentation provided in appendix C).

Table 4 summarises the effectiveness of the interventions as reported by the studies by focusing on the primary outcomes and whether the improvement effected by the intervention was statistically significant. In RCTs, the outcome measures in the intervention arm were compared against the control arm (with the exception of analyses by Keyserling et al., 2014, Stol et al., 2020), whilst in single-arm studies the outcome measures at follow-up were compared against baseline. Various outcome measures were investigated in the reviewed studies. Ten studies (31 %) did not specify a primary outcome. A composite measure concerning mortality and morbidity, i.e. lethal and non-lethal ischemic heart disease events, was reported as a primary outcome in one study. No statistically significant difference between intervention and comparator was found. The most common primary outcome was risk estimated by a CPM. Regarding this outcome, four out of eight studies (50 %) in which the intervention condition and comparator condition were contrasted reported a statistically significant difference in favour of the intervention. These four do not include the mixed results described by Wister et al. (2007). The authors reported a statistically significant difference between the intervention and comparator in the primary prevention group, yet not in the secondary prevention group. Further, five out of seven studies (71 %) in which the comparison regarding risk estimated by a CPM was made to baseline reported a statistically significant improvement. These five do not include the results described by Siren et al. (2016) and by Stol et al. (2020). In the former publication, a statistically significant effect was found in only one of the two CPMs used, noting that the non-significant effect had a p-value of 0.060. In the latter publication, a statistically significant effect was found only after adjusting for aging. Other primary outcomes were biomarkers, anthropometrics, and lifestyle behaviours affecting risk, namely smoking, physical activity, nutrition, medication adherence, and patient activation. In general, the effectiveness of the interventions in terms of statistical significance concerning these outcomes was mixed. Finally, one RCT reported an increase in prescriptions of cardiometabolic medications and an improved detection of cardiometabolic disease, and one RCT reported a statistically significant reduction in decisional regret, contrasting the intervention condition to the comparator condition.

## 4 Discussion

We systematically reviewed existing literature concerning the use of CPMs to personalise lifestyle interventions for the prevention of CVD. Thirty-two studies were included, of which 20 were RCTs (63 %) and 12 were single-arm studies (38 %). Nineteen different CPMs were employed. Notably, in 15 out of 32 interventions (47 %), a version of the FRS was used. Most often, in 18 out of 32 interventions (56 %), the CPM estimates were supplemented with univariable risk factor values. Multiplying the CPM estimate by relative risk estimates of risk factors not included in the model was applied in three studies (9 %), and by relative risk factor estimates of intervention effects in four studies (13 %). Additionally, in three studies (9 %), the CPM estimate was supplemented with a lifestyle score, and in one study (3 %), with a physical examination index. The CPM estimate was used to determine the intervention’s intensity in just five out of 32 studies (16 %). In most studies, 31 out of 32 (97 %), the CPM estimate was used to inform the type of the intervention. Reporting regarding the use of the CPM was deemed lacking detail in 20 out of 32 studies (63 %). No studies were found in which the usage of the CPM was the only experimental variable that varied, therefore, it is inconclusive what effect the CPM in the intervention had. The most commonly used primary outcome were CPM estimates. In four out of eight (50 %) of the analyses comparing the intervention condition and comparator condition, a statistically significant effect in favour of the intervention was reported. Moreover, in five out of seven (71 %) of the analyses with a comparison to baseline, a statistically significant improvement was found.

Considering a quantitative suggestion based on a CPM estimate, both CPMs based on causal or non-causal associations may be used. Remarkably, only a few interventions used the information provided by the CPM estimate in this manner.

Considering the route from a CPM estimate to a suggested qualitative action, different approaches are possible. The CPM estimate may be combined with univariable risk factors to inform the decision. One must be cautious when applying a univariable risk factor cut-off value when a multivariable estimate indicates the individual has a high risk, in order not to exclude those at high risk by having moderately elevated values on multiple risk factors (Alderman, 1993, Jackson et al., 2005). However, at times, it is advised to combine information from a CPM estimate and univariable risk factors, for example, when considering the initiation of antihypertensive medications (e.g., Arnett et al., 2019). Furthermore, when CPMs are used to predict a hypothetical intervention effect to inform the treatment plan, a foundation in causal inference is necessitated (Hernán et al., 2019). A CPM may be combined with relative risk reduction estimates from RCTs, as described by Harrell and Lazzeroni (2017). To derive causal CPMs based on observational data, other techniques have been developed (Lin et al., 2021, Shalit et al., 2017). Additionally, suggested actions may be based on combining non-causal CPMs and decision-curve analyses (Vickers and Elkin, 2006). van der Leeuw and colleagues advocate for using a CPM and accompanying decision-curve analysis for contrasting expected treatment benefits and harms of medications to tailor CVD prevention treatment (van der Leeuw et al., 2014). With the exception of supplementing the CPM estimate with univariable risk factors, these techniques were rarely used in the included studies.

It is worth noting that the information provided by the CPM may be incorporated into a decision-aid. A Cochrane review reported strong evidence indicating that patients using a decision-aid were more knowledgeable of their options and which option they preferred (Stacey et al., 2017). Just six (19 %) of the interventions included used a decision-aid.

Importantly, cultivating a healthy lifestyle is a process. It is likely that the effects of lifestyle interventions diminish over time without sustained efforts. Harting et al. (2006), for example, reported a waning in the decrease of fat intake at 18 months compared to 4 months. Recurrence of unhealthy behaviours should be anticipated in lifestyle interventions, since the patterns are not forgotten, but rather inhibited (Bouton, 2014). A healthy lifestyle is not an end-point you achieve, but a process with ups and downs, requiring continuous efforts. Providing intermediate feedback of the effect of the participant’s behavioural changes on the CPM-based estimated risk may aid the participant to understand this dynamic. This was done in the publications by Edelman et al., 2006, Wister et al., 2007, Khanji et al., 2019, Redfern et al., 2020, and Kwon et al. (2020).

Unfortunately, in none of the studies, the use of a CPM was the only experimental variable that varied. Therefore, it is inconclusive what the effect of the CPM or differential use of the CPM is. Impact studies are needed (Moons et al., 2009). These should not only address a comparison of an intervention condition with a CPM to a comparator condition without a CPM, but also the manner in which a CPM estimate is translated to a suggested action.

Our review was hindered by insufficient detail in reporting concerning the CPM, possibly because the study did not focus on the implementation of the CPM. For example, Ketola et al. (2001) wrote regarding the modified North Karelia risk score “the use of this score assists the staff in recognising, informing and treating high-risk patients” and “an individual multifactorial intervention programme was tailored for each patient according to the risk factor status and needs of the patients”. While this indicates the CPM estimate was used to inform the intervention, it is not clear how. More recently, two extensions to guidelines regarding studies using artificial intelligence have been published: standard protocol items: recommendations for interventional trials - artificial intelligence (SPIRIT-AI) (Cruz Rivera et al., 2020) and consolidated standards of reporting trials – artificial intelligence (CONSORT-AI) (Liu et al., 2020). Whereas the included CPMs may not be considered artificial intelligence, these guidelines do provide guidance to report the application of the CPM in more detail.

Furthermore, it is likely that the listed studies are not exhaustive. Only two databases were searched (PubMed and PsycInfo). However, considering the broadness of these databases, we deem the selected studies do provide a sufficient indication of the usage of CPMs in this type of intervention.

In addition, we acknowledge that statistical significance is likely not the most relevant criterion to determine the intervention’s effectiveness. It has been criticised by statisticians for dichotomising research results based on arbitrary thresholds (Amrhein et al., 2019), and statistically significant effects might fall short from being clinically significant. Moreover, cost-effectiveness is disregarded (van Giessen et al., 2017). Nonetheless, we discussed statistical significance, since we considered the included studies too heterogeneous for meta-analysis, and most often statistical significance played a part in the publication to interpret whether the intervention was effective.

Further, we did not complete a risk of bias assessment. Although this would provide more insight into the primary outcome results, our main aim was to provide an overview of the use of CPMs. A risk of bias assessment would not provide any added value concerning this objective.

Future research on the topic is needed to build better tools and assess their effectiveness, not only in terms of statistical significance, but also examining clinical significance, generalisability, and cost-effectiveness. A well-validated CPM, appropriate to the specific context, may be used as a foundation. Impact analyses are desired, investigating the potential added value of using a CPM versus not using a CPM, and to compare different methods concerning translating a CPM estimate to a suggested action. Furthermore, future research may address conveying the information in an informative and practical manner to the clinician and patient, taking into account the patient’s values. This may encompass the development or fine-tuning of suitable decision-aids. In addition, different channels for the implementation of these tools, such as electronic health records or digital e-coaches hosted in wearable devices or smartphones, may be explored in future studies.

In conclusion, this study presented an overview of the use of CPMs to personalise lifestyle interventions for CVD prevention. Due to the design of the included studies, it is inconclusive what the optimal use of CPMs may be. Therefore we believe there is a need for further research to explore the full potential of using CPMs to personalise lifestyle interventions for the prevention of CVD.

## Funding

This research received funding from the Netherlands Organisation for Scientific Research (NWO): Coronary ARtery disease: Risk estimations and Interventions for prevention and EaRly detection (CARRIER): project nr. 628.011.212.

## Declaration of Competing Interest

The authors declare that they have no known competing financial interests or personal relationships that could have appeared to influence the work reported in this paper.
