# Supplementary material for: Using clinical prediction models to personalise lifestyle interventions for cardiovascular disease prevention: A systematic literature review
Source: Prev Med Rep. 2021 Dec 16;25:101672. doi: 10.1016/j.pmedr.2021.101672 (PMC8800044; doi:10.1016/j.pmedr.2021.101672)
Supplement: Supplementary data 1 [file mmc1.docx]

**Appendix A. Overview database searches**

**Table A.1**

Overview database searches that lead to at least 1 retained item after selection based on title and abstract.

| **Database** | **Search string** | **Applied filters** | **Number of results** | **Number of items screened** |
| --- | --- | --- | --- | --- |
| PubMed | Cardiovascular Diseases/prevention and control[MAJR] AND prediction[Text Word] AND intervention[Text Word] | None | 82 | 82 |
| PubMed | Cardiovascular Diseases/prevention and control[MAJR] AND prediction*[Text Word] AND intervention[Text Word] | None | 91 | 91 |
| PubMed | Cardiovascular Diseases/prevention and control[MAJR] AND risk[Text Word] AND intervention[Text Word] | None | 7 071 | 300 |
| PubMed | Cardiovascular Diseases/prevention and control[MAJR] AND intervention[Text Word] AND (risk scor*[Text Word] OR risk equation[Text Word] OR risk model[Text Word] OR prediction model[Text Word]) | Full Text; Clinical Trial or Randomized Controlled Trial | 105 | 105 |
| PubMed | Cardiovascular Diseases/prevention and control[MAJR] AND intervention[ALL] AND (risk scor*[ALL] OR risk equation[ALL] OR risk model[ALL] OR prediction model[ALL]) | Full Text; Clinical Trial or Randomized Controlled Trial | 183 | 183 |
| PubMed | Cardiovascular Diseases/prevention and control[MAJR] AND cardiovascular[Text Word] AND (risk scor*[ALL] OR risk equation[ALL] OR risk model[ALL] OR prediction model[ALL]) AND (lifestyle[Text Word] OR behavio*[Text Word]) AND intervention[Text Word] | None | 114 | 114 |
| PubMed | Cardiovascular Diseases/prevention and control[MAJR] AND (risk[Text Word] AND (scor*[Text Word] OR factor[Text Word] OR model[Text Word] OR equation[Text Word] OR estimat*[Text Word] OR predict*[Text Word]) AND (lifestyle[Text Word] OR life style[Text Word] OR behavio*[Text Word] OR multi*[Text Word] OR smok*[Text Word] OR diet[Text Word] OR nutrition*[Text Word] OR exercis*[Text Word] OR physical activity[Text Word]) AND (intervention[Text Word] OR counsel*[Text Word] OR motivat*[Text word] OR coach*[Text word])) | Full Text; Clinical Trial or Randomized Controlled Trial | 869 | 869 |
| PsycInfo | DE "Cardiovascular Health" AND (DE "Predictability (Measurement)" OR DE "Prognosis" OR DE "Estimation" OR DE "Risk Assessment" OR DE "Risk Management" OR DE "Risk Factors") | Published date: -20210731; English | 152 | 152 |
| PsycInfo | (DE “Cardiovascular Disorders" OR DE "Cardiovascular Health") AND (DE "Predictability (Measurement)" OR DE "Prognosis" OR DE "Estimation" OR DE "Risk Assessment" OR DE "Risk Factors" OR DE "Risk Management") | Classification Codes: 3360 Health Psychology & Medicine; Published Date: -20210731; English | 121 | 121 |
