# Supplementary material for: Using clinical prediction models to personalise lifestyle interventions for cardiovascular disease prevention: A systematic literature review
Source: Prev Med Rep. 2021 Dec 16;25:101672. doi: 10.1016/j.pmedr.2021.101672 (PMC8800044; doi:10.1016/j.pmedr.2021.101672)
Supplement: Supplementary data 2 [file mmc2.docx]

**Appendix B. Excluded publications based on full-text**

Reason: Absence of a clinical prediction model estimate linked to a suggested action in the intervention.

1. Agewall, S., Wikstrand, J., Samuelsson, O., Persson, B., Andersson, O. K., & Fagerberg, B. (1994). The efficacy of multiple risk factor intervention in treated hypertensive men during long-term follow up. *Journal of Internal Medicine*, *236*(6), 651–659. <https://doi.org/10.1111/j.1365-2796.1994.tb00858.x>
2. Benfari, R. C., McIntyre, K., Eaker, E., Blumberg, S., & Paul, O. (1979). The psychological effects of differential treatment of a high risk sample in a randomized clinical trial. *American Journal of Public Health*, *69*(10), 996–1000. <https://doi.org/10.2105/AJPH.69.10.996>
3. Bove, A. A., Santamore, W. P., Homko, C., Kashem, A., Cross, R., McConnell, T. R., Shirk, G., & Menapace, F. (2011). Reducing cardiovascular disease risk in medically underserved urban and rural communities. *American Heart Journal*, *161*(2), 351–359. <https://doi.org/10.1016/j.ahj.2010.11.008>
4. Cambien, F., Richard, J. L., Ducimetiere, P., Warnet, J. M., & Kahn, J. (1981). The Paris Cardiovascular Risk Factor Prevention Trial: Effects of two years of intervention in a population of young men. *Journal of Epidemiology & Community Health*, *35*(2), 91–97. <https://doi.org/10.1136/jech.35.2.91>
5. Cochrane, T., Davey, R., Iqbal, Z., Gidlow, C., Kumar, J., Chambers, R., & Mawby, Y. (2012). NHS health checks through general practice: Randomised trial of population cardiovascular risk reduction. *BioMed Central Public Health*, *12*(1), 944. <https://doi.org/10.1186/1471-2458-12-944>
6. Connett, J. E., & Stamler, J. (1984). Responses of black and white males to the special intervention program of the Multiple Risk Factor Intervention Trial. *American Heart Journal*, *108*(3), 839–849. <https://doi.org/10.1016/0002-8703(84)90680-X>
7. Danhauer, S. C., Oliveira, B., Myll, J., Berra, K., & Haskell, W. (2004). Successful dietary changes in a cardiovascular risk reduction intervention are differentially predicted by biopsychosocial characteristics. *Preventive Medicine*, *39*(4), 783–790. <https://doi.org/10.1016/j.ypmed.2004.03.003>
8. Dennison, R. A., Feldman, A. L., Usher-Smith, J. A., & Griffin, S. J. (2018). The association between psychosocial factors and change in lifestyle behaviour following lifestyle advice and information about cardiovascular disease risk. *BioMed Central Public Health*, *18*(1), 731. <https://doi.org/10.1186/s12889-018-5655-7>
9. Eaker, E. D., Benfari, R. C., & Reed, R. B. (1982). Coronary risk factor intervention: Characteristics associated with change. *Journal of Clinical Psychology*, *38*(4), 703–717.
10. Fahs, P. S., Pribulick, M., Williams, I. C., James, G. D., Rovynak, V., & Seibold-Simpson, S. M. (2013). Promoting heart health in rural women. *Journal of Rural Health*, *29*(3), 248–257. <https://doi.org/10.1111/j.1748-0361.2012.00442.x>
11. Fakiri, F. E., Bruijnzeels, M. A., Uitewaal, P. J. M., Frenken, R. A. A., Berg, M., & Hoes, A. W. (2008). Intensified preventive care to reduce cardiovascular risk in healthcare centres located in deprived neighbourhoods: A randomized controlled trial. *European Journal of Cardiovascular Prevention & Rehabilitation*, *15*(4), 488–493. <https://doi.org/10.1097/HJR.0b013e3282fceac2>
12. Foraker, R. E., Shoben, A. B., Lopetegui, M. A., Lai, A. M., Payne, P. R. O., Kelley, M., Roth, C., Tindle, H., Schreiner, A., & Jackson, R. D. (2014). Assessment of Life’s Simple 7^TM^ in the primary care setting: The Stroke Prevention in Healthcare Delivery EnviRonmEnts (SPHERE) study. *Contemporary Clinical Trials*, *38*(2), 182–189. <https://doi.org/10.1016/j.cct.2014.03.007>
13. Goessens, B. M. B., Visseren, F. L. J., de Nooijer, J., van den Borne, H. W., Algra, A., Wierdsma, J., & van der Graaf, Y. (2008). A pilot-study to identify the feasibility of an Internet-based coaching programme for changing the vascular risk profile of high-risk patients. *Patient Education and Counseling*, *73*(1), 67–72. <https://doi.org/10.1016/j.pec.2008.06.004>
14. Greaves, C., Gillison, F., Stathi, A., Bennett, P., Reddy, P., Dunbar, J., Perry, R., Messom, D., Chandler, R., Francis, M., Davis, M., Green, C., Evans, P., & Taylor, G. (2015). Waste the Waist: A pilot randomised controlled trial of a primary care based intervention to support lifestyle change in people with high cardiovascular risk. *International Journal of Behavioral Nutrition and Physical Activity*, *12*(1), 1. <https://doi.org/10.1186/s12966-014-0159-z>
15. Grimm, R. H. (1983). The Multiple Risk Factor Intervention Trial in the U.S.: A summary of results at four years in special intervention and usual care men. *Preventive Medicine*, *12*(1), 185–190. <https://doi.org/10.1016/0091-7435(83)90192-5>
16. Gysan, D. B., Millentrup, S., Albus, C., Bjarnason-Wehrens, B., Latsch, J., Gohlke, H., Herold, G., Wegscheider, K., Heming, C., Seyfarth, M., & Predel, H.-G. (2017). Substantial improvement of primary cardiovascular prevention by a systematic score-based multimodal approach: A randomized trial: The PreFord-Study. *European Journal of Preventive Cardiology*, *24*(14), 1544–1554. <https://doi.org/10.1177/2047487317718081>
17. Hanlon, P., McEwen, J., Carey, L., Gilmour, H., Tannahill, C., Tannahill, A., & Kelly, M. (1995). Health checks and coronary risk: Further evidence from a randomised controlled trial. *British Medical Journal*, *311*(7020), 1609–1613. <https://doi.org/10.1136/bmj.311.7020.1609>
18. Hjermann, I. (1988). Strategies for dietary and anti-smoking advice: Practical experiences from the Oslo study. *Drugs*, *36*(Supplement 3), 105–109. <https://doi.org/10.2165/00003495-198800363-00022>
19. Hjermann, I., Holme, I., Byre, K. V., & Leren, P. (1981). Effect of diet and smoking intervention on the incidence of coronary heart disease: Report from the Oslo study group of a randomised trial in healthy men. *Lancet*, *318*(8259), 1303–1310. <https://doi.org/10.1016/S0140-6736(81)91338-6>
20. Holme, I., Hjermann, I., Helgeland, A., & Leren, P. (1985). The Oslo study: Diet and antismoking advice: Additional results from a 5-year primary preventive trial in middle-aged men. *Preventive Medicine*, *14*(3), 279–292. <https://doi.org/10.1016/0091-7435(85)90057-X>
21. Holme, I., Retterstøl, K., Norum, K. R., & Hjermann, I. (2016). Lifelong benefits on myocardial infarction mortality: 40-year follow-up of the randomized Oslo diet and antismoking study. *Journal of Internal Medicine*, *280*(2), 221–227. <https://doi.org/10.1111/joim.12485>
22. Joshi, R., Agrawal, T., Fathima, F., Usha, T., Thomas, T., Misquith, D., Kalantri, S., Chidambaram, N., Raj, T., Singamani, A., Hegde, S., Xavier, D., Devereaux, P., Pais, P., Gupta, R., & Yusuf, S. (2019). Cardiovascular risk factor reduction by community health workers in rural India: A cluster randomized trial. *American Heart Journal*, *216*, 9–19. <https://doi.org/10.1016/j.ahj.2019.06.007>
23. Kornitzer, M., De Backer, G., Dramaix, M., & Thilly, C. (1980). The Belgian Heart Disease Prevention Project: Modification of the coronary risk profile in an industrial population. *Circulation*, *61*(1), 18–25. <https://doi.org/10.1161/01.CIR.61.1.18>
24. Kouwenhoven-Pasmooij, T. A., Djikanovic, B., Robroek, S. J. W., Helmhout, P., Burdorf, A., & Hunink, M. G. M. (2015). Design and baseline characteristics of the PerfectFit study: A multicenter cluster-randomized trial of a lifestyle intervention in employees with increased cardiovascular risk. *BioMed Central Public Health*, *15*(1), 715. <https://doi.org/10.1186/s12889-015-2059-9>
25. Kouwenhoven-Pasmooij, T. A., Robroek, S. J. W., Kraaijenhagen, R. A., Helmhout, P. H., Nieboer, D., Burdorf, A., & Myriam Hunink, M. G. (2018). Effectiveness of the blended-care lifestyle intervention ‘PerfectFit’: A cluster randomised trial in employees at risk for cardiovascular diseases. *BioMed Central Public Health*, *18*(1), 766. <https://doi.org/10.1186/s12889-018-5633-0>
26. Lopez-Gonzalez, A. A., Aguilo, A., Frontera, M., Bennasar-Veny, M., Campos, I., Vicente-Herrero, T., Tomas-Salva, M., De Pedro-Gomez, J., & Tauler, P. (2015). Effectiveness of the Heart Age tool for improving modifiable cardiovascular risk factors in a Southern European population: A randomized trial. *European Journal of Preventive Cardiology*, *22*(3), 389–396. <https://doi.org/10.1177/2047487313518479>
27. Man, M.-S., Rick, J., & Bower, P. (2015). Improving recruitment to a study of telehealth management for long-term conditions in primary care: Two embedded, randomised controlled trials of optimised patient information materials. *Trials*, *16*(1), 309. <https://doi.org/10.1186/s13063-015-0820-0>
28. Margolis, L. H., Richmond, A., Brown, T., & Jackson, S. (2003). Working with African American small businesses to implement an on-site cardiovascular health program. *Journal of Health Care for the Poor and Underserved*, *14*(3), 331–340. <https://doi.org/10.1353/hpu.2010.0563>
29. Meland, E., Lærum, E., & Ulvik, R. J. (1997). Effectiveness of two preventive interventions for coronary heart disease in primary care. *Scandinavian Journal of Primary Health Care*, *15*(1), 57–63. <https://doi.org/10.3109/02813439709043432>
30. Meland, E., Mæland, J. G., & Lærum, E. (1999). The importance of self-efficacy in cardiovascular risk factor change. *Scandinavian Journal of Public Health*, *27*(1), 11–17. <https://doi.org/10.1177/14034948990270011001>
31. Mendivil, C. O., Cortés, E., Sierra, I. D., Ramírez, A., Molano, L. M., Tovar, L. E., Vargas, C., Granados, N., & Pérez, C. E. (2006). Reduction of global cardiovascular risk with nutritional versus nutritional plus physical activity intervention in Colombian adults. *European Journal of Cardiovascular Prevention & Rehabilitation*, *13*(6), 947–955. <https://doi.org/10.1097/01.hjr.0000219114.48285.7a>
32. Miettinen, T. A., Huttunen, J. K., Naukkarinen, V., Strandberg, T., Mattila, S., Kumlin, T., & Sarna, S. (1985). Multifactorial primary prevention of cardiovascular diseases in middle-aged men: Risk factor changes, incidence, and mortality. *Journal of the American Medical Association*, *254*(15), 2097–2102.
33. Molokhia, M., & Oakeshott, P. (2000). A pilot study of cardiovascular risk assessment in Afro-Caribbean patients attending an inner city general practice. *Family Practice*, *17*(1), 60–62. <https://doi.org/10.1093/fampra/17.1.60>
34. Multiple Risk Factor Intervention Trial Research Group. (1982). Multiple Risk Factor Intervention Trial: Risk factor changes and mortality results. *Journal of the American Medical Association*, *248*(12), 1465–1477.
35. Multiple Risk Factor Intervention Trial Research Group. (1986). Coronary heart disease death, nonfatal acute myocardial infarction and other clinical outcomes in the Multiple Risk Factor Intervention Trial. *American Journal of Cardiology*, *58*(1), 1–13. <https://doi.org/10.1016/0002-9149(86)90232-8>
36. O’Malley, P. G., Feuerstein, I. M., & Taylor, A. J. (2003). Impact of electron beam tomography, with or without case management, on motivation, behavioral change, and cardiovascular risk profile: A randomized controlled trial. *Journal of the American Medical Association*, *289*(17), 2215. <https://doi.org/10.1001/jama.289.17.2215>
37. Pająk, A., Wolfshaut-Wolak, R., Doryńska, A., Jankowski, P., Fornal, M., Grodzicki, T., Jennings, C., Kawecka-Jaszcz, K., Kotseva, K., Pająk, K., & Wood, D. (2020). Longitudinal effects of a nurse-managed comprehensive cardiovascular disease prevention program for hospitalized coronary heart disease patients and primary care high-risk patients. *Kardiologia Polska*, *78*(5), 429–437. <https://doi.org/10.33963/KP.15273>
38. Patterson, T. L., Sallis, J. F., Nader, P. R., Rupp, J. W., McKenzie, T. L., Roppe, B., & Bartok, P. W. (1988). Direct observation of physical activity and dietary behaviors in a structured environment: Effects of a family-based health promotion program. *Journal of Behavioral Medicine*, *11*(5), 447–458. <https://doi.org/10.1007/BF00844838>
39. Racette, S. B., Deusinger, S. S., Inman, C. L., Burlis, T. L., Highstein, G. R., Buskirk, T. D., Steger-May, K., & Peterson, L. R. (2009). Worksite Opportunities for Wellness (WOW): Effects on cardiovascular disease risk factors after 1 year. *Preventive Medicine*, *49*(2–3), 108–114. <https://doi.org/10.1016/j.ypmed.2009.06.022>
40. Robinson, N., Miller, A., Wilbur, J., & Fogg, L. (2018). Subjective versus objective estimated cardiovascular disease risk and adherence to physical activity in African American women. *Journal of Cardiovascular Nursing*, *33*(2), 111–117. <https://doi.org/10.1097/JCN.0000000000000437>
41. Rodríguez Cristóbal, J. J., Alonso-Villaverde Grote, C., Travé Mercadé, P., M^a^ Pérez Santos, J., Peña Sendra, E., Muñoz Lloret, A., Fernández Pérez, C., & Bleda Fernández, D. (2012). Randomised clinical trial of an intensive intervention in the primary care setting of patients with high plasma fibrinogen in the primary prevention of cardiovascular disease. *BioMed Central Research Notes*, *5*(1), 126. <https://doi.org/10.1186/1756-0500-5-126>
42. Saffi, M. A. L., Polanczyk, C. A., & Rabelo-Silva, E. R. (2014). Lifestyle interventions reduce cardiovascular risk in patients with coronary artery disease: A randomized clinical trial. *European Journal of Cardiovascular Nursing*, *13*(5), 436–443. <https://doi.org/10.1177/1474515113505396>
43. Salisbury, C., O’Cathain, A., Thomas, C., Edwards, L., Gaunt, D., Dixon, P., Hollinghurst, S., Nicholl, J., Large, S., Yardley, L., Fahey, T., Foster, A., Garner, K., Horspool, K., Man, M.-S., Rogers, A., Pope, C., & Montgomery, A. A. (2016). Telehealth for patients at high risk of cardiovascular disease: Pragmatic randomised controlled trial. *British Medical Journal*, i2647. <https://doi.org/10.1136/bmj.i2647>
44. Shefer, G., Silarova, B., Usher-Smith, J., & Griffin, S. (2016). The response to receiving phenotypic and genetic coronary heart disease risk scores and lifestyle advice – A qualitative study. *BioMed Central Public Health*, *16*(1), 1221. <https://doi.org/10.1186/s12889-016-3867-2>
45. Shlay, J. C., Barber, B., Mickiewicz, T., Maravi, M., Drisko, J., Estacio, R., Gutierrez, G., & Urbina, C. (2011). Reducing cardiovascular disease risk using patient navigators, Denver, Colorado, 2007-2009. *Preventing Chronic Disease*, *8*(6), A143.
46. Silarova, B., Sharp, S., Usher-Smith, J. A., Lucas, J., Payne, R. A., Shefer, G., Moore, C., Girling, C., Lawrence, K., Tolkien, Z., Walker, M., Butterworth, A., Di Angelantonio, E., Danesh, J., & Griffin, S. J. (2019). Effect of communicating phenotypic and genetic risk of coronary heart disease alongside web-based lifestyle advice: The INFORM randomised controlled trial. *Heart*, *105*(13), 982–989. <https://doi.org/10.1136/heartjnl-2018-314211>
47. Singh, R. B., Sharma, V. K., Gupta, R. K., & Singh, R. (1992). Nutritional modulators of lipoprotein metabolism in patients with risk factors for coronary heart disease: Diet and moderate exercise trial. *Journal of the American College of Nutrition*, *11*(4), 391–398. <https://doi.org/10.1080/07315724.1992.10718242>
48. Sorensen, M. (1997). Maintenance of exercise behavior for individuals at risk for cardiovascular disease. *Perceptual and Motor Skills*, *85*(3), 867–880. <https://doi.org/10.2466/pms.1997.85.3.867>
49. Soureti, A., Hurling, R., Murray, P., van Mechelen, W., & Cobain, M. (2010). Evaluation of a cardiovascular disease risk assessment tool for the promotion of healthier lifestyles. *European Journal of Cardiovascular Prevention & Rehabilitation*, *17*(5), 519–523. <https://doi.org/10.1097/HJR.0b013e328337ccd3>
50. Stefanich, C. A., Witmer, J. M., Young, B. D., Benson, L. E., Penn, C. A., Ammerman, A. S., Garcia, B. A., Jilcott, S. B., & Etzel, R. A. (2005). Development, adaptation, and implementation of a cardiovascular health program for Alaska native women. *Health Promotion Practice*, *6*(4), 472–481. <https://doi.org/10.1177/1524839904263725>
51. Storm, V., Dörenkämper, J., Reinwand, D. A., Wienert, J., De Vries, H., & Lippke, S. (2016). Effectiveness of a web-based computer-tailored multiple-lifestyle intervention for people interested in reducing their cardiovascular risk: A randomized controlled trial. *Journal of Medical Internet Research*, *18*(4), e78. <https://doi.org/10.2196/jmir.5147>
52. Tekkeşin, A. İ., Hayıroğlu, M. İ., Çinier, G., Özdemir, Y. S., İnan, D., Yüksel, G., Pay, L., Parsova, K. E., Vatanoğlu, E. G., Şeker, M., Durak, F., & Gürkan, K. (2021). Lifestyle intervention using mobile technology and smart devices in patients with high cardiovascular risk: A pragmatic randomised clinical trial. *Atherosclerosis*, *319*, 21–27. <https://doi.org/10.1016/j.atherosclerosis.2020.12.020>
53. Turner, B. J., Hollenbeak, C. S., Liang, Y., Pandit, K., Joseph, S., & Weiner, M. G. (2012). A randomized trial of peer coach and office staff support to reduce coronary heart disease risk in African-Americans with uncontrolled hypertension. *Journal of General Internal Medicine*, *27*(10), 1258–1264. <https://doi.org/10.1007/s11606-012-2095-4>
54. Vale, M. J., Jelinek, M. V., Best, J. D., Dart, A. M., Grigg, L. E., Hare, D. L., Ho, B. P., Newman, R. W., & McNeil, J. J. (2003). Coaching patients on Achieving Cardiovascular Health (COACH): A multicenter randomized trial in patients with coronary heart disease. *Archives of Internal Medicine*, *163*(22), 2775. <https://doi.org/10.1001/archinte.163.22.2775>
55. Villablanca, A. C., Beckett, L. A., Li, Y., Leatherwood, S., Gill, S. K., Giardina, E.-G. V., Taylor, A. L., Barron, C., Foody, J. M., Haynes, S., & D’Onofrio, G. (2010). Outcomes of comprehensive heart care programs in high-risk women. *Journal of Women’s Health*, *19*(7), 1313–1325. <https://doi.org/10.1089/jwh.2009.1426>
56. Voogdt-Pruis, H. R., Gorgels, A. P. M., van Ree, J. W., van Hoef, E. F. M., & Beusmans, G. H. M. I. (2010). Patient perceptions of nurse-delivered cardiovascular prevention: Cross-sectional survey within a randomised trial. *International Journal of Nursing Studies*, *47*(10), 1237–1244. <https://doi.org/10.1016/j.ijnurstu.2010.02.013>
57. Vučak, J. (2014). Is a targeted and planned GP intervention effective in cardiovascular disease prevention? A randomized controlled trial. *Medical Science Monitor*, *20*, 1180–1187. <https://doi.org/10.12659/MSM.890242>
58. Westlake, C., Evangelista, L. S., Strömberg, A., Ter-Galstanyan, A., Vazirani, S., & Dracup, K. (2007). Evaluation of a web-based education and counseling pilot program for older heart failure patients. *Progress in Cardiovascular Nursing*, *22*(1), 20–26. <https://doi.org/10.1111/j.0889-7204.2007.05703.x>
59. Zhang, H., Jiang, Y., Nguyen, H. D., Poo, D. C. C., & Wang, W. (2017). The effect of a Smartphone-Based Coronary Heart Disease Prevention (SBCHDP) programme on awareness and knowledge of CHD, stress, and cardiac-related lifestyle behaviours among the working population in Singapore: A pilot randomised controlled trial. *Health and Quality of Life Outcomes*, *15*(1), 49. <https://doi.org/10.1186/s12955-017-0623-y>

Reason: Related to an included, more relevant publication.

1. Aadahl, M., von Huth Smith, L., Toft, U., Pisinger, C., & Jørgensen, T. (2011). Does a population-based multifactorial lifestyle intervention increase social inequality in physical activity? The Inter99 study. *British Journal of Sports Medicine*, *45*(3), 209–215. <https://doi.org/10.1136/bjsm.2009.064840>
2. Badenbroek, I. F., Stol, D. M., Nielen, M. M., Hollander, M., Kraaijenhagen, R. A., de Wit, G. A., Schellevis, F. G., & de Wit, N. J. (2014). Design of the INTEGRATE study: Effectiveness and cost-effectiveness of a cardiometabolic risk assessment and treatment program integrated in primary care. *BioMed Central Family Practice*, *15*(1), 90. <https://doi.org/10.1186/1471-2296-15-90>
3. Baumann, S., Toft, U., Aadahl, M., Jørgensen, T., & Pisinger, C. (2015). The long-term effect of screening and lifestyle counseling on changes in physical activity and diet: The Inter99 Study – A randomized controlled trial. *International Journal of Behavioral Nutrition and Physical Activity*, *12*(1), 33. <https://doi.org/10.1186/s12966-015-0195-3>
4. Bender, A. M., Jørgensen, T., & Pisinger, C. (2019). Higher mortality in women living in high-participation areas of a population-based health check and lifestyle intervention study. *International Journal of Public Health*, *64*(1), 107–114. <https://doi.org/10.1007/s00038-018-1179-2>
5. Cox, J. L., Carr, B., Vallis, T. M., Szpilfogel, C., & O’Neill, B. J. (2011). A novel approach to cardiovascular health by optimizing risk management (ANCHOR): A primary prevention initiative examining the impact of health risk factor assessment and management on cardiac wellness. *Canadian Journal of Cardiology*, *27*(6), 809–817. <https://doi.org/10.1016/j.cjca.2011.04.013>
6. Hempler, N. F., Krasnik, A., Pisinger, C., & Jørgensen, T. (2012). The relationship between changes in health behaviour and initiation of lipid-lowering and antihypertensive medications in individuals at high risk of ischaemic heart disease. *BioMed Central Public Health*, *12*(1), 626. <https://doi.org/10.1186/1471-2458-12-626>
7. Krones, T., Keller, H., Becker, A., Sönnichsen, A., Baum, E., & Donner-Banzhoff, N. (2010). The theory of planned behaviour in a randomized trial of a decision aid on cardiovascular risk prevention. *Patient Education and Counseling*, *78*(2), 169–176. <https://doi.org/10.1016/j.pec.2009.06.010>
8. løkkegaard, T., Andersen, J. s., Jacobsen, R. k., Badsberg, J. H., Jørgensen, T., & Pisinger, C. (2015). Psychological consequences of screening for cardiovascular risk factors in an un-selected general population: Results from the Inter99 randomised intervention study. *Scandinavian Journal of Public Health*, *43*(1), 102–110. <https://doi.org/10.1177/1403494814557886>
9. Pisinger, C., Glümer, C., Toft, U., von Huth Smith, L., Aadahl, M., Borch-Johnsen, K., & Jørgensen, T. (2008). High risk strategy in smoking cessation is feasible on a population-based level. The Inter99 study. *Preventive Medicine*, *46*(6), 579–584. <https://doi.org/10.1016/j.ypmed.2008.02.026>
10. Pisinger, C., Toft, U., Aadahl, M., Glümer, C., & Jørgensen, T. (2009). The relationship between lifestyle and self-reported health in a general population. *Preventive Medicine*, *49*(5), 418–423. <https://doi.org/10.1016/j.ypmed.2009.08.011>
11. Sheridan, S. L., Draeger, L. B., Pignone, M. P., Sloane, P. D., Samuel-Hodge, C., Finkelstein, E. A., Gizlice, Z., Vu, M. B., Gitterman, D. P., Bangdiwala, S. I., Donahue, K. E., Evenson, K., Ammerman, A. S., & Keyserling, T. C. (2013). Designing and implementing a comparative effectiveness study of two strategies for delivering high quality CHD prevention: Methods and participant characteristics for the Heart to Health study. *Contemporary Clinical Trials*, *36*(2), 394–405. <https://doi.org/10.1016/j.cct.2013.07.013>
12. Thomsen, T. F., Davidsen, M., Ibsen, H., Jorgensen, T., Jensen, G., & Borch-Johnsen, K. (2001). A new method for CHD prediction and prevention based on regional risk scores and randomized clinical trials; PRECARD and the Copenhagen risk score. *European Journal of Cardiovascular Prevention & Rehabilitation*, *8*(5), 291–297. <https://doi.org/10.1177/174182670100800508>
13. Toft, U., Kristoffersen, L., Ladelund, S., Ovesen, L., Lau, C., Borch-Johnsen, K., Pisinger, C., & Jørgensen, T. (2008). The impact of a population-based multi-factorial lifestyle intervention on changes in long-term dietary habits. *Preventive Medicine*, *47*(4), 378–383. <https://doi.org/10.1016/j.ypmed.2008.05.013>
14. Toft, U. N., Kristoffersen, L. H., Aadahl, M., von Huth Smith, L., Pisinger, C., & Jorgensen, T. (2007). Diet and exercise intervention in a general population—Mediators of participation and adherence: The Inter99 study. *European Journal of Public Health*, *17*(5), 455–463. <https://doi.org/10.1093/eurpub/ckl262>
15. Toft, U., Pisinger, C., Aadahl, M., Lau, C., Linneberg, A., Ladelund, S., Kristoffersen, L., & Jørgensen, T. (2009). The impact of a population-based multi-factorial lifestyle intervention on alcohol intake. *Preventive Medicine*, *49*(2–3), 115–121. <https://doi.org/10.1016/j.ypmed.2009.06.007>
16. von Huth Smith, L., Ladelund, S., Borch-Johnsen, K., & Jørgensen, T. (2008). A randomized multifactorial intervention study for prevention of ischaemic heart disease (Inter99): The long-term effect on physical activity. *Scandinavian Journal of Public Health*, *36*(4), 380–388. <https://doi.org/10.1177/1403494807085313>

Reason: Not focused on lifestyle.

1. Cykert, S., Keyserling, T. C., Pignone, M., DeWalt, D., Weiner, B. J., Trogdon, J. G., Wroth, T., Halladay, J., Mackey, M., Fine, J., In Kim, J., & Cene, C. (2020). A controlled trial of dissemination and implementation of a cardiovascular risk reduction strategy in small primary care practices. *Health Services Research*, *55*(6), 944–953. <https://doi.org/10.1111/1475-6773.13571>
