# Supplementary material for: Using clinical prediction models to personalise lifestyle interventions for cardiovascular disease prevention: A systematic literature review
Source: Prev Med Rep. 2021 Dec 16;25:101672. doi: 10.1016/j.pmedr.2021.101672 (PMC8800044; doi:10.1016/j.pmedr.2021.101672)
Supplement: Supplementary data 3 [file mmc3.docx]

**Appendix C. Argumentation concerning insufficient detail in reporting**

**Table C.1**

Argumentation concerning insufficient detail in reporting.

| **Study name (if any)** | **Citation** | **Argumentation** |
| --- | --- | --- |
| - | (Ketola et al., 2001) | Insufficient detail to determine how CPM was used exactly; no English publication available concerning development of CPM |
| - | (Edelman et al., 2006) | Missing reference CPM |
| Hartslag Limburg | (Harting et al., 2006) | Refer to guidelines, application concerning lifestyle intervention not clear |
| Simon Fraser heart health report card system | (Wister et al., 2007) | Insufficient detail to determine how letter grading system was developed |
| ARRIBA-Herz | (Krones et al., 2008) | No references provided concerning relative risk estimates |
| Healthy hearts | (Richardson et al., 2008) | Refer to guidelines, application not clear; website in reference no longer active |
| IMPALA | (Koelewijn-van Loon et al., 2009) | Decision-aid not available, link CPM estimates and options to reduce risk not clear |
| COHRT | (Nolan et al., 2011) | Insufficient detail to determine how CPM was used exactly |
| HAPPY / MYCLIC NL | (Hofstra et al., 2011; Yousuf et al., 2019) | Insufficient detail construction lifestyle score, insufficient detail how lifestyle score and CPM inform advice |
| Fremantle primary prevention study | (Brett et al., 2012) | Unclear what is meant by advice attempt to improve CPM estimate |
| IEHPS | (Liu et al., 2015) | Insufficient detail to determine how CPM was used exactly, physical examination index not defined |
| INTEGRATE (pilot) | (van den Brekel-Dijkstra et al., 2016) | Reference of CPM provides insufficient information about the development of the CPM |
| - | (Siren et al., 2016) | Refer to guidelines, application unclear; no English publication available concerning development of CPM |
| CHARLAR | (Krantz et al., 2017) | Refer to guidelines, application not clear |
| DECADE | (Tinsel et al., 2018) | No references provided concerning relative risk estimates |
| ACTIVATE | (Oddone et al., 2018) | Reference of CPM provides insufficient information about the development of the CPM |
| HAPPY / MYCLIC: AZM | (Yousuf et al., 2019) | Insufficient detail construction lifestyle score, insufficient detail how lifestyle score and CPM inform advice |
| HAPPY / MYCLIC: London | (Khanji et al., 2019; Yousuf et al., 2019) | Insufficient detail construction lifestyle score, insufficient detail how lifestyle score and CPM inform advice |
| - | (Kavita et al., 2020) | Refer to guidelines, application unclear |
| CONNECT | (Redfern et al., 2020) | Missing reference CPM |
| ACTIVATE = a coaching by telephone intervention for veterans and care team engagement; ARRIBA-Herz = Aufgabe gemeinsam definieren, Risiko subjektiv, Risiko objektiv, Information über Präventionsmöglichkeiten, Bewertung der Präventionsmöglichkeiten und Absprache über weiteres Vorgehen – Herz (define task together, subjective risk, objective risk, information about prevention options and agreement on further action - heart); AZM =Academisch Ziekenhuis Maastricht (Maastricht University Medical Centre+); CHARLAR = community heart health actions for Latinos at risk; COHRT = community outreach heart health and risk reduction trial; CONNECT = consumer navigation of electronic cardiovascular tools; CPM = clinical prediction model; DECADE = decision-aid, action planning, and follow-up support for patients to reduce the 10-year risk of CVD; HAPPY = heart attack prevention program for you; IEHPS = individualised electronic healthcare prescription software; IMPALA = improving patient adherence to lifestyle advice; MyCLIC = my cardiac lifestyle intervention coach; NL = Netherlands | | |
